# The significance of ErbB2/3 in the conversion of induced pluripotent stem cells into cancer stem cells

**DOI:** 10.1038/s41598-022-04980-y

**Published:** 2022-02-17

**Authors:** Ghmkin Hassan, Maram H. Zahra, Akimasa Seno, Masaharu Seno

**Affiliations:** 1 Department of Biotechnology and Drug Discovery, Graduate School of Interdisciplinary Science and Engineering in Health Systems, Okayama University, 3.1.1 Tsushima-Naka, Kita, Okayama 700-8530 Japan; 2 Department of Genomic Oncology and Oral Medicine, Graduate School of Biomedical and Health Science, Hiroshima University, Hiroshima, 734-8553 Japan; 3 The Laboratory of Natural Food and Medicine, Co., Ltd., Okayama, 700-8530 Japan

**Keywords:** Cancer microenvironment, Cancer stem cells

## Abstract

Cancer stem cells (CSCs) are suggested to be responsible for drug resistance and aggressive phenotypes of tumors. Mechanisms of CSC induction are still under investigation. Our lab has established a novel method to generate CSCs from iPSCs under a cancerous microenvironment mimicked by the conditioned medium (CM) of cancer-derived cells. Here, we analyzed the transcriptome of CSCs, which were converted from iPSCs with CM from pancreatic ductal adenocarcinoma cells. The differentially expressed genes were identified and used to explore pathway enrichment. From the comparison of the CSCs with iPSCs, genes with elevated expression were related to the ErbB2/3 signaling pathway. Inhibition of either ErbB2 with lapatinib as a tyrosine kinase inhibitor or ErbB3 with TX1-85-1 or siRNAs arrested cell proliferation, inhibited the in vitro tumorigenicity, and lead to loss of stemness in the converting cells. The self-renewal and tube formation abilities of cells were also abolished while CD24 and Oct3/4 levels were reduced, and the MAPK pathway was overactivated. This study shows a potential involvement of the ErbB2/ErbB3 pathway in CSC generation and could lead to new insight into the mechanism of tumorigenesis and the way of cancer prevention.

## Introduction

Cancer is still one of the major health problems in the world. The cancer initiation mechanisms have been focused to understand the process of tumorigenesis and develop efficient treatments^1,2^. Cancer stem cells (CSCs) are considered to be responsible for the cancer initiation with self-renewal and differentiation abilities as well as drug resistance and cancer relapse but the rare cell subpopulation in tumor tissues is still substantial for the availability^3,4^.

Nowadays, CSCs have been isolated and characterized in almost all types of cancers. The chronic inflammation and imbalanced microenvironment are suggested as tumor driving events by genetically transforming or epigenetically converting normal stem cells into CSCs^4–6^. Revealing exact mechanisms for cancer initiation is still challenging because it requires observation of early tumorigenesis events. The existing models, which are produced by introducing foreign genes and mutations, usually fail to capture these events^7^. Moreover, CSCs isolated from patient tumors already underwent genetic changes altering the modes of signaling pathways^3,6^. Therefore, the conversion of pluripotent stem cells into CSCs provides a powerful tool to investigate the CSC generation process. In this context, our lab has established a method to convert induced pluripotent stem cells (iPSCs) or embryonic stem cells (ESCs) into CSCs under a microenvironment mimicking cancer-inducing niche^8^. The method employs conditioned media (CM) from cancer-derived cells that are rich in growth factors, chemokines, and tissue-specific factors mimicking cancer-inducing microenvironments. With this method, iPSCs were converted into CSCs with high malignant tumorigenicity and metastatic potential. We have successfully generated models for liver, pancreatic, lung, and breast CSCs^8–12^. The pancreatic CSC model was established with CM from two different pancreatic cancer cell lines PK8 and KLAM-1. The established CSCs showed malignancy with the signature of pancreatic duct adenocarcinoma^10^.

ERBB subfamily belongs to the receptor tyrosine kinase (RTK) superfamily with four members of ErbB1/EGFR, ErbB2, ErbB3, and ErbB4. ErbB ligands contain a conserved EGF motif and they are responsible for the activation of ErbBs. The ligands induce homo- or heterodimerization between ErbBs activating intracellular signal transduction^13,14^. ErbB signaling pathways regulate cellular functions and its dysregulations result in dysfunction of cells and development of diseases. The ErbB3 that has weak tyrosine kinase activity forms a heterodimer with ErbB2 that does not have a known ligand. The neuregulin-1 (Nrg-1) and neuregulin-2 (Nrg-2) are ligands for ErbB3^15,16^. The high expression levels of ErbB subfamily members were linked to tumor grades, relapse, and resistance to treatments. The co-expression of ErbB2 and ErbB3 was reported in different types of cancers. Recent data suggest that ErbB2/ErbB3 heterodimer has pivotal roles in tumorigenesis and CSC proliferation, survival, and self-renewal^17–21^. The inactivation of ErbB2 was shown to decrease ErbB3 tyrosine phosphorylation^17^. MEK/ERK signaling pathways is one of the pathways existing downstream of ErbB receptors. The inhibition of MEK/ERK signaling was shown to promote self-renewal and pluripotency of mouse ESCs^22^.

In this study, the role of the ErbB2/ErbB3 heterodimer was evaluated in the conversion of mouse iPSCs (miPSCs) into CSCs. By the treatment of iPSCs with CM derived from cancer cells, miPSCs gained CSC characteristics. The transcriptome from CSCs, which were converted from iPSCs, revealed elevated expression of genes related to the ErbB signaling pathway. Inhibition of either ErbB2 or ErbB3 was shown to inhibit the in vitro tumorigenicity, self-renewal, and stemness of converted cells.

## Results

### The pancreatic CSC model upregulated the expression of genes in the ErbB2/ErbB3 signal pathway

**Figure 1:**
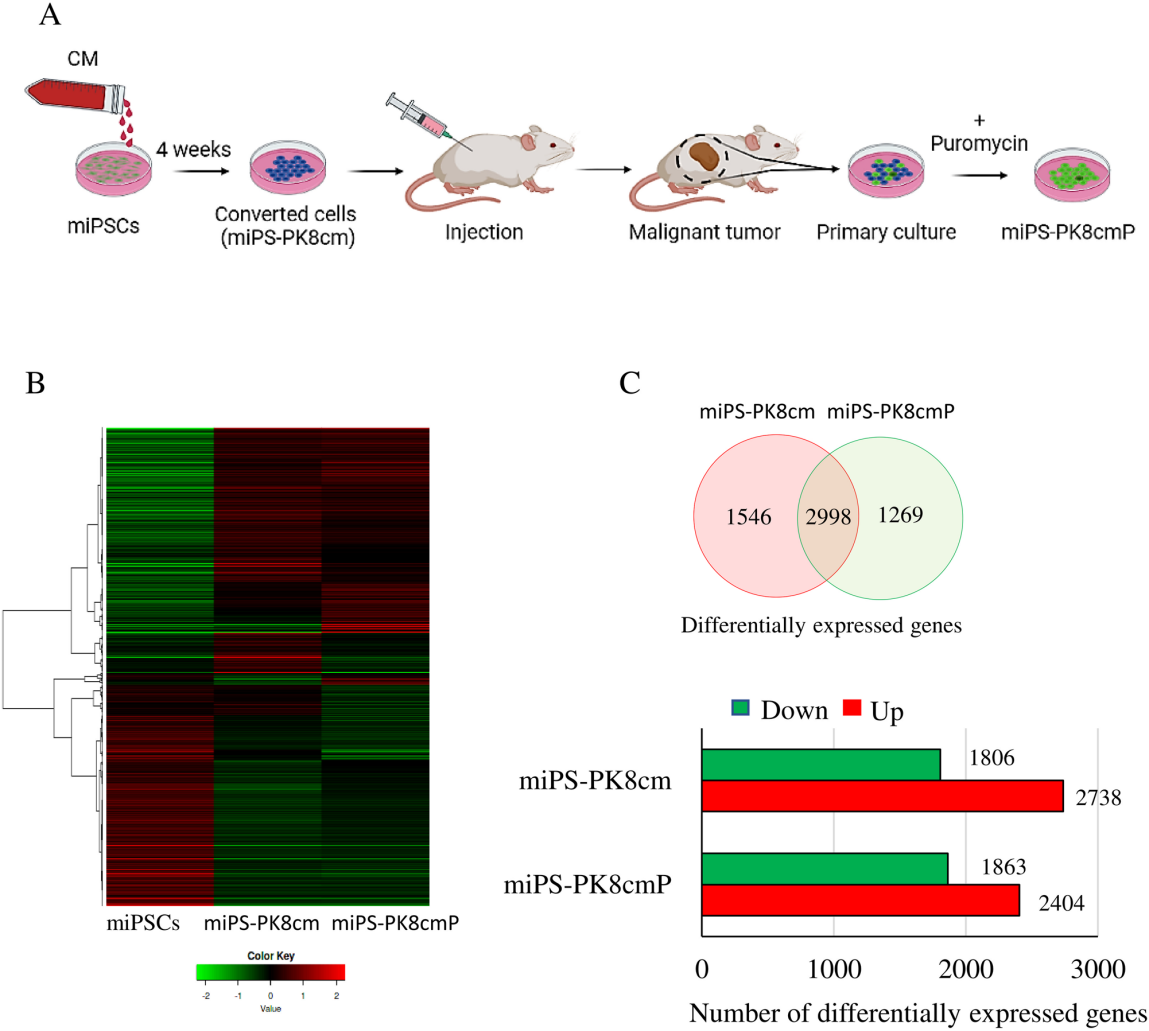
Analysis of transcriptomes of CSCs converted from miPSCs by RNA-seq. (**A**) Schematic chart of conversion of miPSCs into CSCs, converted cells were prepared by culturing miPSCs in 50% CM, then cells were transplanted into mice and CSCs were isolated from the primary culture of the tumors. (**B)** A heat map of gene expression in miPSCs, miPS-PK8cm, and miPS-PK8cmP cells. (**C)** A Venn diagram showing the differentially expressed genes in miPS-PK8cm and miPS-PK8cmP cells when compared with those in miPSCs and the bar graph depicting the number of up- and downregulated genes in miPS-PK8cm and miPS-PK8cmP cells when compared with those in miPSCs.

We have previously established a pancreatic CSC model converted from miPSCs using conditioned media (CM) from human pancreatic cancer cell line PK8 cells^10^. The converted cells when injected into immunodeficient mice, developed malignant tumors exhibiting CSC characteristics. The primary culture of the tumor developed in the pancreas also showed the expression of stemness and CSC markers as well as the markers of pancreatic duct adenocarcinoma and the ability of metastasis^10^. The CSCs were efficiently selected with the resistance to puromycin and GFP expression since the original miPSCs carried genes for puromycin resistance and GFP downstream of the Nanog promoter (Fig. 1A). The resultant cells converted from miPSC were designated as miPS-PK8cm cells and the CSCs were designed as miPS-PK8cmP cells. The RNA from these cells were subjected to the RNA-seq and the gene expression patterns were compared as a heat map (Fig. 1B).

**Figure 2:**
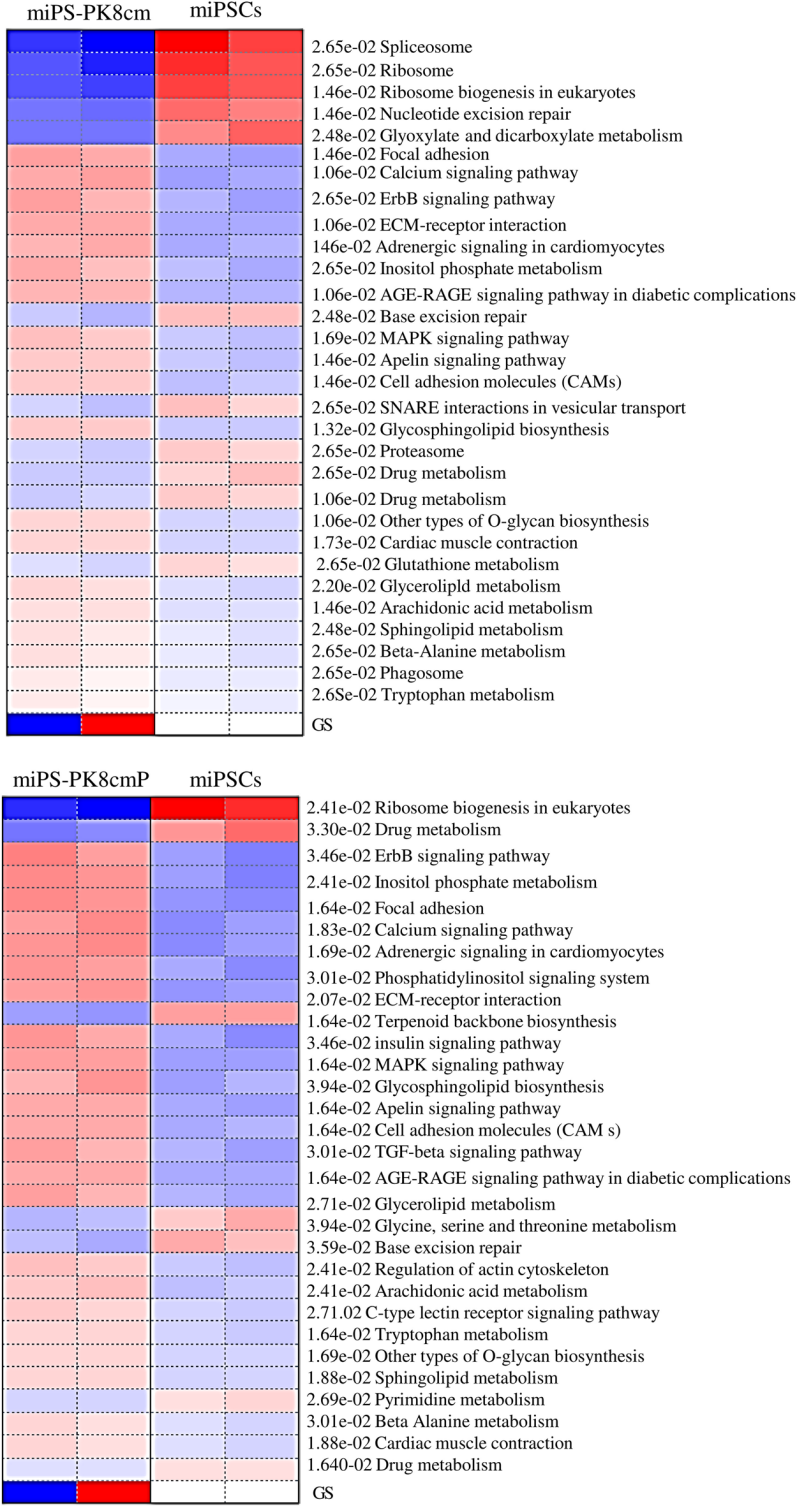
The top enriched pathways in CSCs converted from miPSCs. The top enriched pathways for the upregulated genes in miPS-PK8cm and miPS-PK8cmP cells compared with miPSCs. Red and blue indicates activated and suppressed pathways, respectively.

The number of genes expressed differentially from miPSCs was found to be 4544 and 4267 for miPS-PK8cm and miPS-PK8cmP cells, respectively. The number of upregulated genes was 2738 and 2404 in miPS-PK8cm and miPS-PK8cmP cells, respectively (Fig. 1C). The pathway analyses with Parametric Gene Set Enrichment Analysis (PGSEA) showed the top activated pathways enriched in miPS-PK8cm cells when compared with miPSCs were ‘focal adhesion’, ‘calcium signaling pathway’ and ‘ErbB signaling pathway’. Similarly, the top activated pathways enriched in miPS-PK8cmP cells were ‘ErbB signaling pathway’, ‘inositol phosphate metabolism’ and ‘focal adhesion’ (Fig. 2). From these data, we hypothesized that the ErbB signaling pathway could be responsible for the conversion of iPSCs into CSCs under the pancreatic microenvironment reflected by CM of PK8 cells. Next, we compared expression levels of different members of the ErbB subfamily and their ligands. The RNA-seq data revealed that the expression of ErbB2, ErbB3, Nrg-1, and Nrg-2 was the highest among the members (Supplementary Table 1). Moreover, we analyzed the gene expression of ErbB2 and 3 during the two weeks of conversion process. The data showed that one week treatment of cells with CM significantly elevated expression levels of ErbB2 and 3 (Supplementary Fig. 1). We next performed the pairwise comparison between PK8 cells and normal pancreas cells to investigate upregulated secreted factors produced by PK8 and could be responsible for the conversion. The data showed that more than 30 genes of inflammatory-related secretory factors were overexpressed in PK8 cells. These genes included interleukins (IL) such as IL36G, IL11, IL18, IL1B, IL1A and IL23A, chemokines such as C-X-C motif ligand 1 (CXCL1), CXCL6, CXCL8, CXCL10, CXCL11 and C–C motif chemokine ligand 20 (CCL20),  and growth factors such as NRG1, EREG, TGFA and BMP2 (Supplementary Fig. 2, Supplementary data 2). Among these genes, NRG1, the ligand for ErbB3, was found to be the highest overexpressed gene. Finally, we also analyzed the sequences of ErbB2 and ErbB2 mRNA translated into amino acids in miPS-PK8cm cells and compared them with reference sequences. The data showed that amino acid sequences in miPS-PK8cm cells are completely identical with those translated from mErbB3 (accession NM_010153) and mErbB2 (accession NM_0010038173). (Supplementary data 3 and 4). These data suggested a potential role of cellular stimulation through ErbB2 and ErbB3, which act as a heterodimeric membrane receptor of intracellular signaling during the conversion process and this role is not related with genetic alterations.

### Inhibition of ErbB2 or ErbB3 suppressed the conversion of miPSCs into CSCs

**Figure 3:**
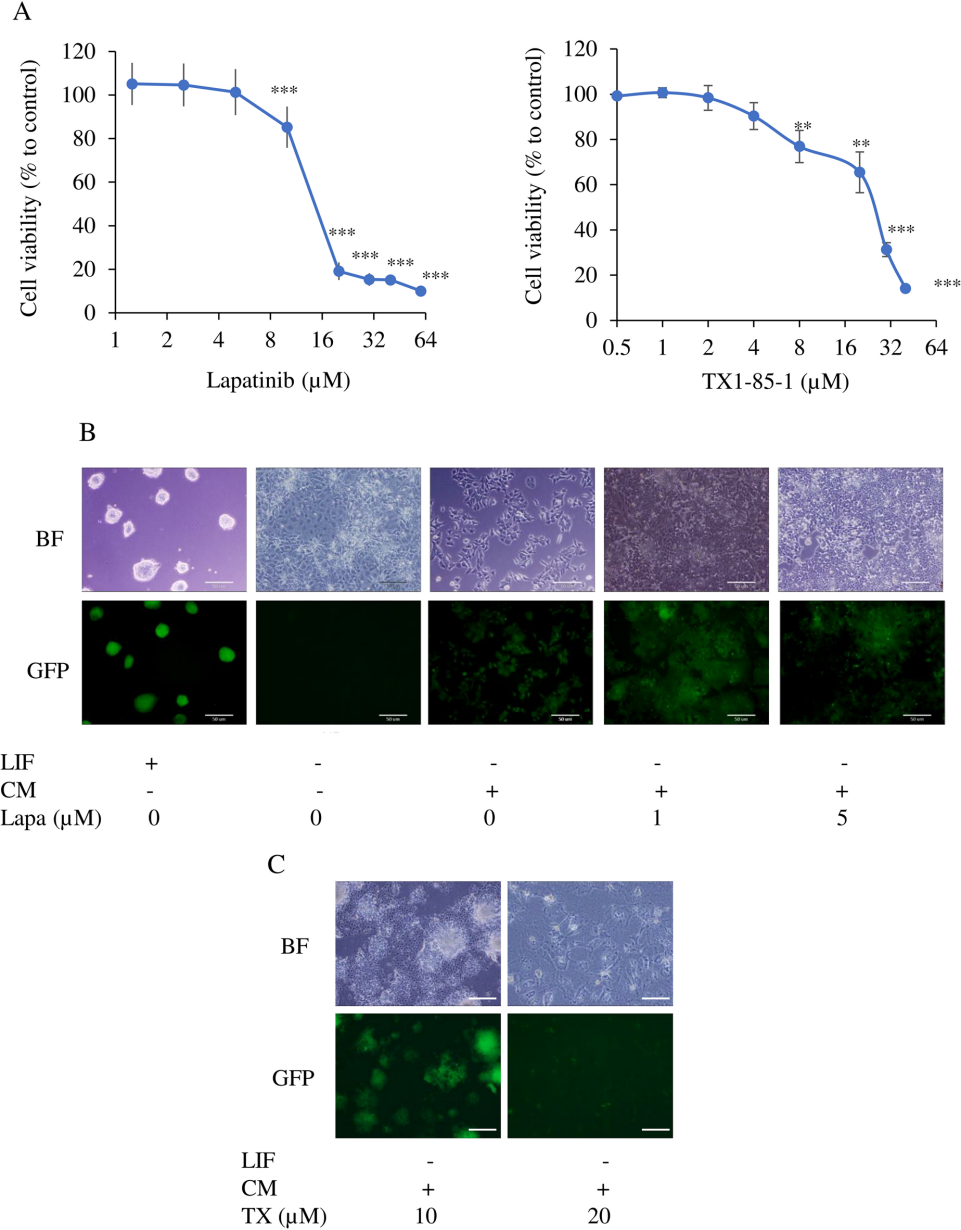
The effect of lapatinib and TX1-85-1 on miPSCs in the presence of CM of PK8 cells. (**A**) Cell viability evaluated by MTT assay. **, *p* < 0.005; ***, *p* < 0.001. (**B,C)** Representative bright-field (BF) and fluorescence (GFP) images of cells after one week of culturing in different conditions with or without LIF, CM, and lapatinib (lapa) at 1 or 5 µM or TX1-85-1 (TX) at 10 or 20 µM. Scale bars = 50 µm.

To assess the role of the ErbB2/ErbB3 signal in the conversion process, the hypothesis raised in the previous section, lapatinib as a tyrosine kinase inhibitor for ErbB2 and TX1-85-1 as ErbB3 inhibitor were used to interrupt tyrosine kinases. The MTT assay showed that the IC_50_ of lapatinib on miPSCs was approximately 12 µM while IC_50_ of TX1-85-1 was approximately 25 µM (Fig. 3A). The miPSCs are routinely cultured with leukemia inhibitory factor (LIF) to maintain stemness and miPSCs will start differentiation and stop growth namely fail to survive without LIF. The miPSCs cultured without LIF lost GFP expression and did not survive through passages. In contrast, in the presence of the CM of PK8 cells, miPSCs maintained GFP expression and kept proliferation without LIF. The CM with lapatinib in 1 µM or 5 µM appeared to maintain GFP while enhancement of differentiation in adhesive cells with 5 µM was observed (Fig. 3B). A portion of cells cultured with CM and 10 µM TX1-85-1 maintained GFP and differentiated cells were shown as GFP negative cells in the adhesive cell culture condition. The cells lost GFP expression completely and differentiated with 20 µM TX1-85-1 and could not survive beyond one week of the treatment (Fig. 3C).

**Figure 4:**
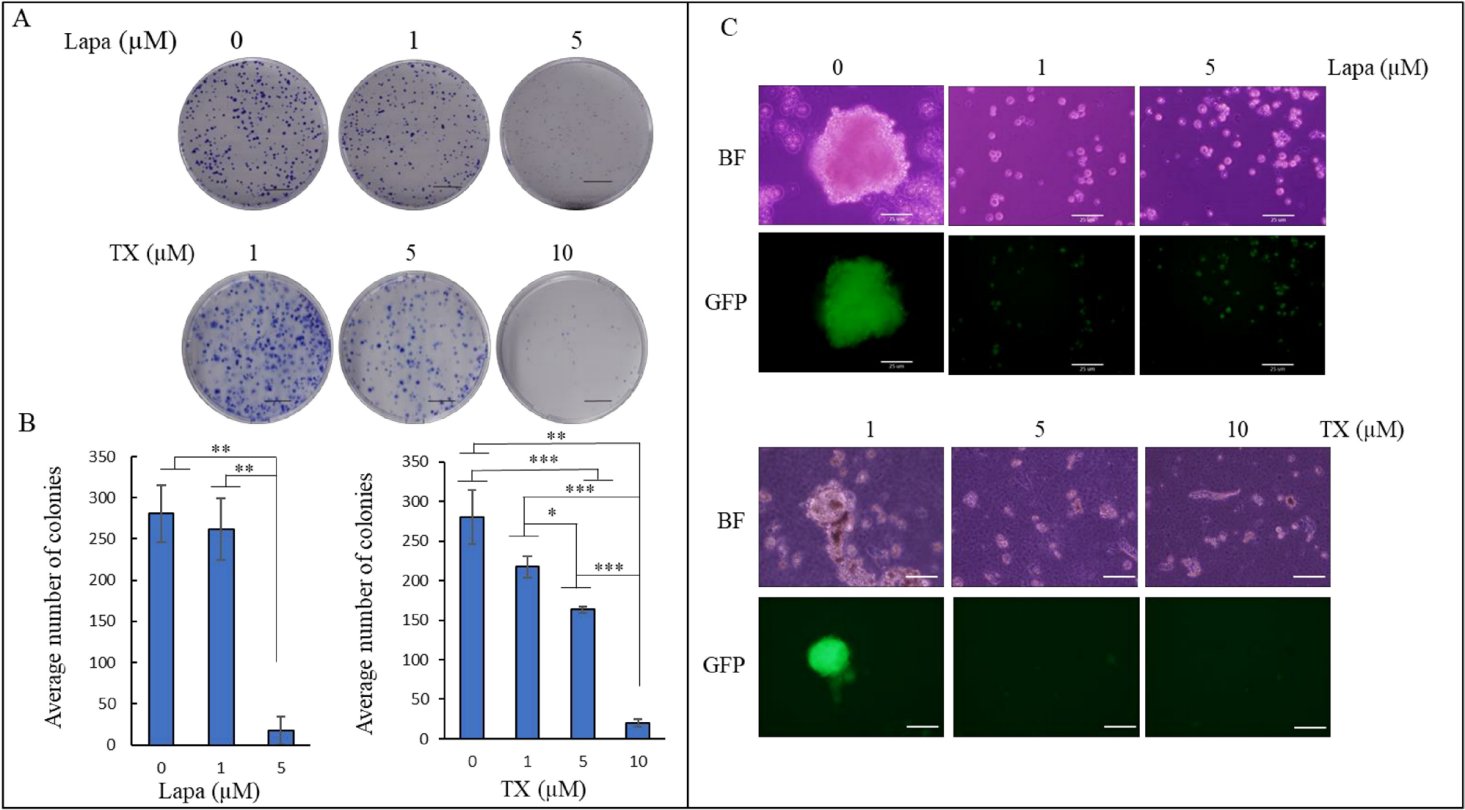
The effect of lapatinib and TX1-85-1 on the self-renewal ability of miPSCs cultured in CM. (**A**) Representative images of colonies formed by miPSCs cultured in different conditions. Scale bar = 1 cm. (**B)** The bar graph depicts the average number of colonies after one week of culturing miPSCs in the CM supplemented with either 1 or 5 µM of lapatinib or with 1, 5 or 10 µM of TX1-85-1. Data were from three independent experiments (n = 3). (**C)** Representative bright-field (BF) and fluorescence (GFP) images of a sphere formed by miPSCs cultured in the same treatment conditions as described above show inhibition of sphere formation ability by lapatinib and TX1-85-1. Scale bars = 25 µm. Experiments were repeated three times.

Our group has demonstrated that iPSCs converted into CSCs maintain self-renewal and colony-forming abilities when culture in CM of cancer cells^8,23^. The effect of the lapatinib and TX1-85-1 on the formation of colonies and spheroids was assessed during the conversion. The miPSCs cultured in the presence of 1 and 5 µM lapatinib showed a decrease in the number of colonies (Fig. 4A). While the decrease with 1 µM was insignificant, 5 µM exhibited a significant decrease in the number of colonies from 250 down to 17 (Fig. 4B). Cells cultured with TX1-85-1 showed a decrease in colony numbers in a dose-dependent manner. The average colony number were 217, 163 and 20 for 1, 5 and 10 µM TX1-85-1, respectively (Fig. 4A,C). One and 5 µM of lapatinib, 5 and 10 µM TX1-85-1 also inhibited the spheroid formation ability of the miPSCs cultured under low attachment culture conditions in serum-free media while the miPSCs cultured with CM maintained spheroid formation ability (Fig. 4C).

### The effect of lapatinib on tube formation ability of miPSCs when converted into CSCs

**Figure 5:**
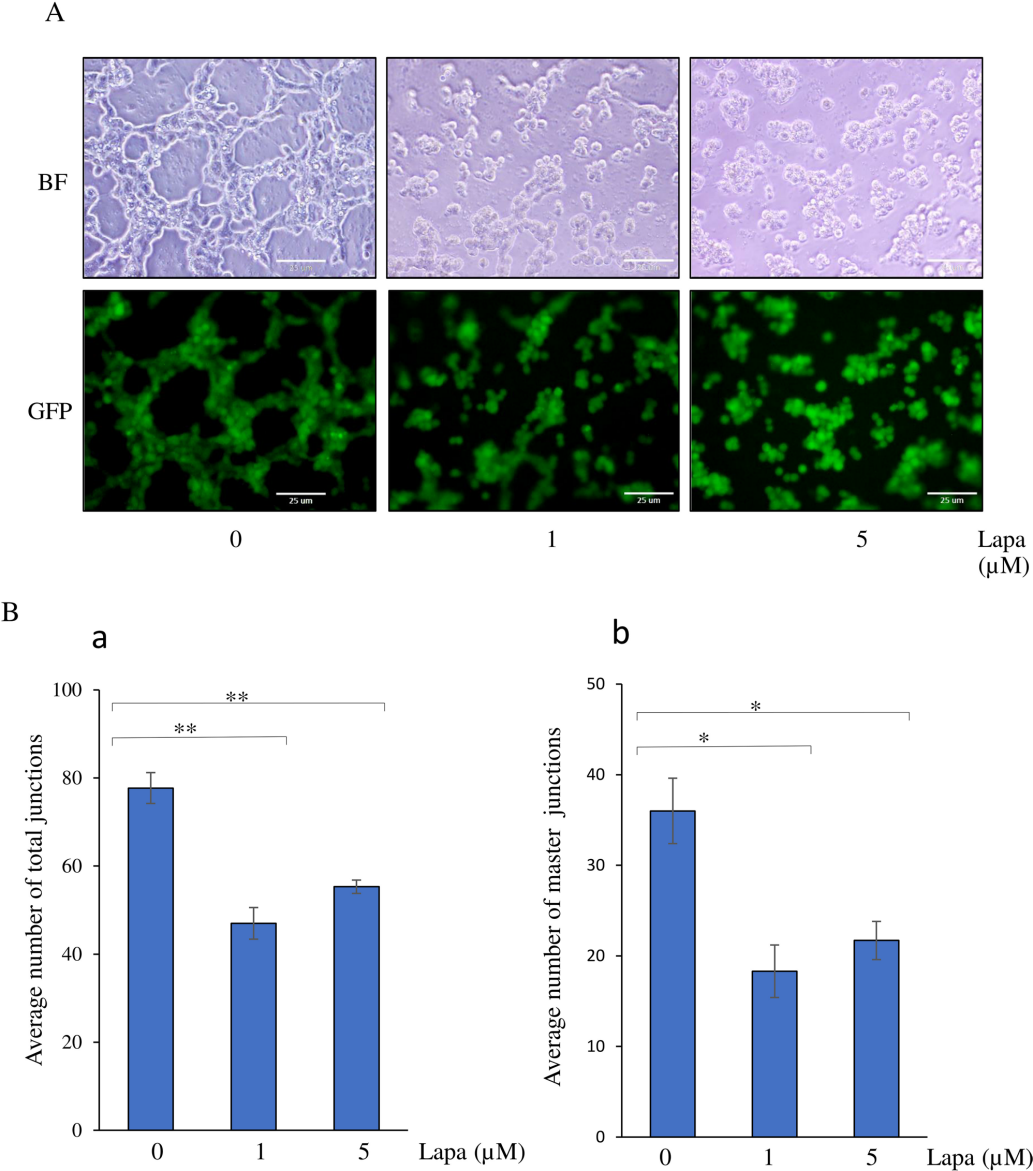
The effect of lapatinib on tube formation ability of miPSCs cultured in CM. (**A**) Representative bright field and fluorescence images of tube-like structures of miPSCs cultured in different conditions of treatment exhibiting the inhibition of formation of tube-like structures in the presence of lapatinib. Scale bars = 25 µm. (**B)** Bar graphs showing the average number of total junctions (**a**) and master junctions (**b**) formed by cells. *, *p* < 0.005; **, *p* < 0.001.

Tumor angiogenesis is one of the essential cancer hallmarks. Previously we demonstrated in several reports that CSCs converted from miPSCs promoted tumor angiogenesis with their ability to form tube-like structures in the presence of type IV collagen and to differentiate into CD31 positive cells^11^. Thus, we assessed the effect of the lapatinib on the tube formation ability of miPSCs cultured in CM with or without lapatinib for one week. When transferred to Matrigel-coated well plates, miPSCs exhibited tube-like structures, which were inhibited with 1 and 5 µM of lapatinib (Fig. 5A). In the presence of lapatinib, numbers of ‘total junctions’ and ‘master junctions’ formed by iPSCs were significantly reduced when compared with those cultured in only CM (Fig. 5B).

### Inhibition of ErbB2 or ErbB3 enhanced the activation of Erk and attenuated stemness during conversion

**Figure 6:**
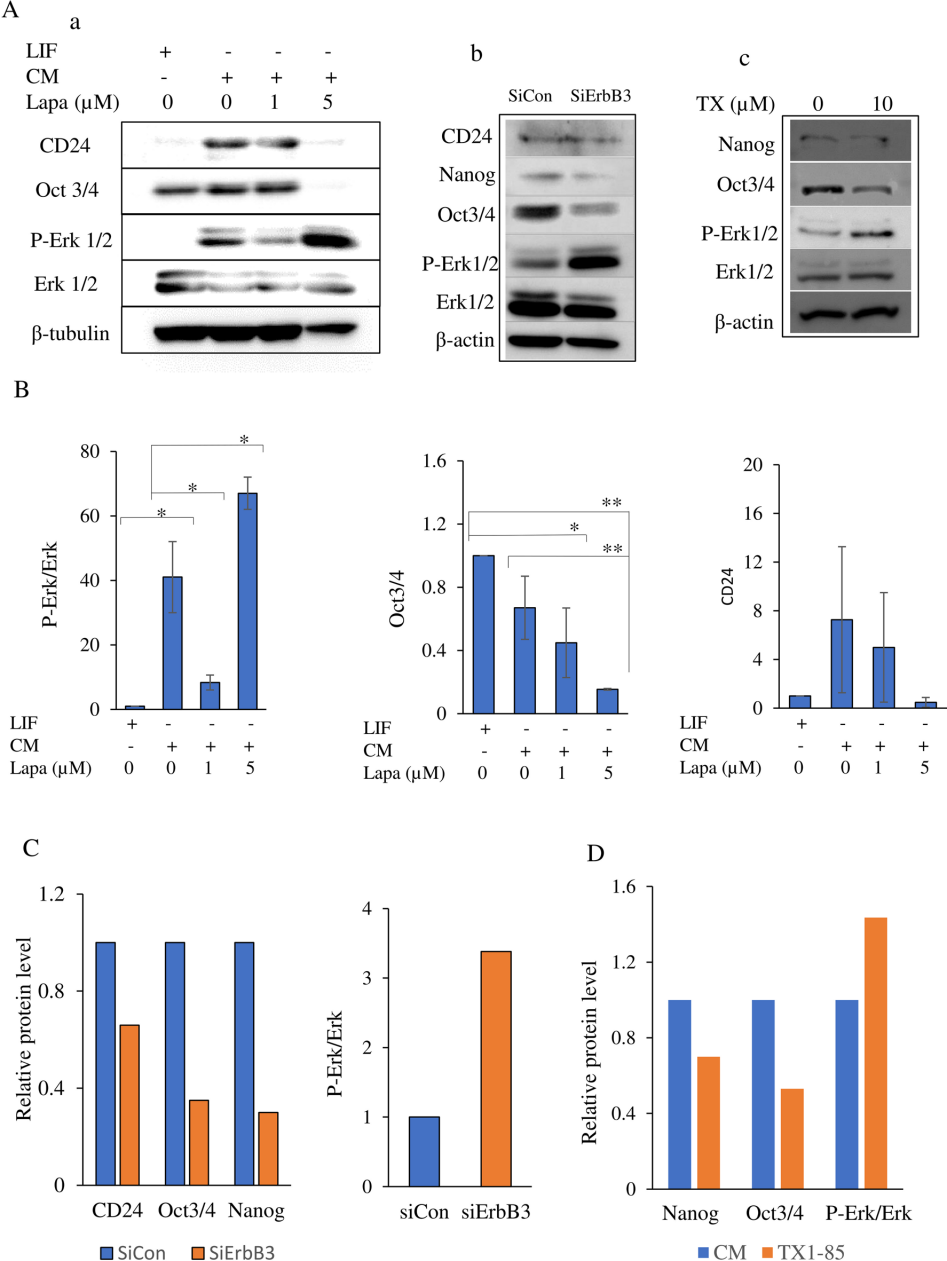
Inhibition of ErbB2 or ErbB3 modulated Erk activation and decreased stemness. (**A**) Western blot analysis of miPSCs cultured in different conditions, in the presence of lapatinib (**a**), by inhibition of ErbB3 with siRNA (**b**) or in the presence of TX1-85-1 (**c**). Antibodies against β-tubulin, β-actin, Erk1/2, phospho-Erk1/2, Oct3/4, Nanog and CD24 were used as primary antibodies. (**B–D)** Bar graphs represent relative protein levels normalized with β-tubulin or β-actin levels in the presence of lapatinib (**B**), by inhibition of ErbB3 with siRNA (**C**) or in the presence of TX1-85-1 (**D**). siCon: Negative siRNA control, siErbB3: ErbB3 siRNA. Results in (**Aa**)) are representative of three experiments and values in B are mean ± SE. *, *p* < 0.05; **, *p* < 0.001.

To investigate the role of the ErbB2 and ErbB3 signaling in the conversion process, the effects of lapatinib and TX1-85-1 were assessed on miPSCs for the acquisition of CSC characteristics during conversion. The western blot showed that miPSCs cultured in CM maintained the Oct3/4 level while CD24, as a CSC marker, slightly increased when compared with that in miPSCs cultured with LIF. On the other hand, the level of Oct3/4 and CD24 were maintained in miPSCs cultured in the presence of 1 µM lapatinib. The Oct3/4 and CD24 levels were dramatically decreased in the presence of 5 µM of lapatinib compared to the levels in cells cultured without lapatinib (Fig. 6Aa,B). The data also showed overactivation of Erk along with attenuation of stemness in the miPSCs cultured in the presence of 5 µM of lapatinib when compared with the level in miPSCs cultured without lapatinib. One µM lapatinib decreased Erk activation in miPSCs cultured in CM when compared to that without lapatinib (Fig. 6Aa,B). Inhibition of ErbB3 with small interfering RNA (siRNA) or 10 µM TX1-85-1 decreased Oct3/4 and Nanog levels and overactivated Erk. Targeting ErbB3 with siRNA also showed a decrease in CD24 level compared with negative siRNA control. (Fig. 6Ab,c,C,D).

## Discussion

Different intracellular signal pathways and their upstream molecules such as tyrosine kinase receptors like ErbBs regulate the tumorigenicity of CSCs^24^. The deregulation of tyrosine kinases by either genetic and/or epigenetic modifications is considered the common cause of cancer initiation in many types of cancers. The dysregulation was linked to stemness, differentiation, and induction of metastasis in different cancer models^25^.

Studies on CSCs could substantially be difficult due to the very small rates of existence in tumors rendering the obstacles of obtaining or maintaining^26^. Moreover, the existing CSC models fail to capture early events of tumor initiation and their mechanisms in most cases. This issue reduces the opportunities to identify the mechanisms of tumorigenesis and to reveal the importance of searching for new models of CSCs as well as cancer initiation^27,28^. The conversion of normal stem cells into CSCs offers a promising and unique tool to overcome these obstacles. Accordingly, our lab has established new CSC models by converting iPSCs and embryonic stem cells into CSCs with different tissue specificities^8,9,11^. With these models, our previous analyses proposed that CSCs could be induced by epigenetic events of DNA hypomethylation in CpG islands^29,30^.

Previously, the ErbB2/ErbB3 heterodimer has been described as an oncogenic unit in breast cancer^17^. The heterodimerization of ErbB2 and ErbB3 could be responsible for the conversion process as suggested by the RNA-seq analysis in this study. Generally, an ErbB ligand is considered to induce either homo- or heterodimerization of receptors triggering phosphorylation of the half moiety of a dimer. The gene expression profiles suggested that the expression of both Nrg-1 and Nrg-2 genes in miPSCs increased during the conversion where the expression level of Nrg-2 was more than that of Nrg-1. This enhancement of expression went along with the elevation of expression levels of ErbB2 and ErbB3 proposing a possible autocrine loop for Nrg-1 and Nrg-2 in the conversion. The CM of cancer cells contains variable inflammatory-related growth factors, cytokines, and other molecules, which would act as a mimic of the cancer-initiating microenvironment by changing signaling pathways. The autocrine loop of Nrg-1/2 with ErbB2/ErbB3 heterodimer elucidates the activation of downstream pathways and their crosstalk with one another as a key event in the conversion of normal stem cells into CSCs under inflammatory microenvironments such as pancreatitis. The lapatinib as tyrosine kinase inhibitor specific to ErbB2, TX1-85-1 as ErbB3 inhibitor and siRNA targeting ErbB3 exhibited the ability to hinder the conversion process. The self-renewal and in vitro tumorigenic abilities of iPSCs were inhibited by these inhibitors when assessed by sphere and colony formation assays, respectively while the CM maintained these abilities. The inhibition of self-renewal and colony formation abilities by 5 µM lapatinib, 10 µM TX1-85-1 and ErbB3’s siRNA went along with a decrease in the level of Oct3/4 as a stemness marker and a suppression of the elevation of CD24 as a CSC marker. These changes were also going along with overactivation of the Erk indicating the activation of RAF/MEK/ERK cascade repressing CSC generation. Not only the pathway alone but also its crosstalk contributes to tumorigenicity and self-renewal to maintain CSCs. Different signaling pathways can be activated through ErbBs depending on the type of ligand and receptor dimers. These pathways are RAS/RAF/MEK/ERK, JAK-STAT, Nck/PAK, and Phosphatidylinositol 3-kinase (PI3K)/ protein kinase B (AKT) including phospholipase Cγ. ErbBs could be transactivated by several mechanisms involving crosstalk between ErbBs and other pathways such as JAK-STAT in which phosphorating Jak downstream of the activated growth hormone receptors can activate ErbBs^31,32^. Interestingly, in converted cells, Jak1 and several chemokines and interleukins such as Ccl2, Il17d, Cxcl1, Cxcl12, and Ccl7 were found among differentially upregulated genes (Supplementary Table 1). The integrins and voltage-gated Ca_2_^+^ channels can also transactivate ErbB receptors through phosphorylation by other kinases^32^. The PGSEA showed that those pathways were among the top enriched ones in converted cells while genes related to them were found to be differentially upregulated (Fig. 2, Supplementary Table 1).

The Erk is a downstream target of tyrosine kinases including ErbB receptors^33^. While CM showed a small activation of Erk compared with miPSCs which could be because of some cell differentiation since CM contains a variety of factors or due to cross-talking between different pathways, the 5 µM lapatinib, 10 µM TX1-85-1 and ErbB3’s siRNA treatment resulted in overactivation of Erk compared with CM and cell's stemness was attenuated. The activation of MEK/ERK was linked to cancer stemness attenuation. Chan et al. showed that the downregulation of protein methyltransferase 6 (PRMT6) suppresses MEK/ERK signaling in hepatocellular carcinoma (HCC) and promotes cancer stemness and tumor-initiating properties of HCC cells^34^. Also, a small level of RAF/MEK/ERK activation was observed by inactivation of P53 in advanced prostate cancer patients^35,36^. Finally, exposure normal stem cells, iPSCs, to CM which mimics chronic inflammatory conditions convert them into CSCs by epigenetic and signaling pathway changes. Here, we showed that ErbB2/ErbB3 heterodimer could have essential roles in this process when iPSCs treat with CM from pancreatic cancer cells.

In conclusion, ErbB2/ErbB3 heterodimer activation could be a key point in the conversion of normal stem cells into CSCs under abnormal conditions enriched with different growth and pro-inflammatory factors keeping continuous stimulation of receptors. These data could give insights into CSC generation under an imbalanced homeostatic microenvironment in the pancreas which could be used to predict or eliminate cancer initiation by understanding the mechanisms of CSC generation.

## Material and methods

### Cell culture

The miPSCs (iPS-MEF-Ng-20D-17, Lot No. 012, Riken Cell Bank, Japan) were maintained and cultured as mentioned previously^10^. Converted cells and CSCs designated as miPS-PK8cm and miPS-PK8cmP were previously established by the conversion of miPSCs cells in the presence of conditioned medium from human pancreatic cancer cell line PK8 cells (miPS-PK8cm) and the primary culture of the tumor formed in Balb/c nude mice (miPS -PK8cmP)^10^. The miPS-PK8cmP cells were maintained in 0.1% gelatin-coated 60 mm-dishes with miPS media consisting of DMEM, 15% fetal bovine serum (FBS) (Thermo Fisher Scientific, MA), 2 mM L-glutamine (Nacalai Tesque, Japan), 0.1 mM non-essential amino acid (NEAA), (Thermo Fisher Scientific, MA), and 0.1 mM 2-mercaptoethanol (β-ME) (Sigma-Aldrich, MO). The media was supplemented with 100 U/ml penicillin and 100 µg/ml streptomycin (Wako, Japan).

The human pancreatic cancer cell line PK8 cells (RIKEN, Japan) were cultured in RPMI-1640 (Wako, Japan) containing 10% FBS supplemented with 100 U/ml penicillin and 100 µg/ml streptomycin. When the cells reached 70–80% confluence, the media was changed to RPMI-1640 containing 5% FBS to prepare the CM. The CM was collected after 48 h of adding 5% FBS supplemented media, centrifuged at 1000xg for 10 min, and the supernatant was then filtered through 0.45 μm filters (Sartorius, Göttingen, Germany). The Lapatinib (GW-572016) was purchased from (Selleck, Japan) and TX1-85-1 was from (Cayman Chemical, USA). The miPSCs were cultured either in miPS media with1000 U/mL LIF, 50% CM without LIF, 50% CM and 1 or 5 µM lapatinib without LIF, or 50% CM and 10 or 20 µM TX1-85-1 without LIF.

### RNA sequencing (RNA-seq) and bioinformatic analysis

Total RNA was isolated from cells using RNAiso Plus (Takara Bio, Japan) and RNA was treated with DNase I (Promega, WI) according to manufacturer instructions. The RNA integrity was determined by Agilent Bioanalyzer 2100. The Sequencing libraries were prepared using NEBNext Ultra II RNA Library Prep Kit (New England Biolabs, MA) for samples with RNA Integrity Number (RIN) more than 7. The 150-bp paired sequencing was performed with Novaseq6000 (Illumina, CA). The RNA-seq data was further analyzed on the Galaxy platform < usegalaxy.org > . The Tophat was used to align reads to the mouse reference genome while the Cufflinks used to assemble transcripts and assess their abundances. Then, Cuffmerge, Cuffquant, and Cuffnrorm were used to merge, quantify, and normalize transcripts, respectively. The heat map was generated and the upregulated genes in differential expressed genes (DEGs) with fold change ≥ 2 and false positive rate (FDR) < 0.1 were further used for Parametric Gene Set Enrichment Analysis (PGEEA) with integrated Differential Expression and Pathway analysis (iDEP) < http://bioinformatics.sdstate.edu/idep93/ > . For analysis of microarray data, microarray data for PK8 cells was obtained from the GSE141247 dataset and that for normal pancreas cells was obtained from GSE71729. Pairwise comparison was conducted using Exatls tool < https://lgsun.irp.nia.nih.gov/exatlas/ > with false discovery rate (FDR) ≤ 0.0001 and fold change ≥ 2.

### Quantitative reverse transcription PCR (RT–qPCR)

Total RNA was extracted as same as above and treated with RNase-Free DNase (Promega, CA). One microgram of RNA was reverse transcribed by GoScript™ Reverse Transcriptase (Promega, CA) and RT–qPCR reactions were performed with LightCycler® 480 SYBR Green I Master Mix (Roche, Switzerland) and with primers sets enlisted in Supplementary Table 2.

### MTT assay

The miPSCs were seeded in 96-well plates at 5000 cells/well density with miPS media supplemented with 50% CM and incubated at 37 °C and 5% CO_2_. After 24 h of seeding, cells were incubated with a different concentration of lapatinib or TX1-85-1 and after 24 h of incubation, cell viability was evaluated by thiazolyl blue tetrazolium blue (MTT, Sigma-Aldrich, MO). MTT solution at 0.5 mg/mL concentration was added into wells and plates were incubated for 4 h at 37 °C in 5% CO_2_. After incubation, media were discarded, and 100 µL of DMSO was used to dissolve the formazan crystals and the absorbance was measured at 570 nm by the MTP-800 Lab microplate reader (Corona Electric, Japan). The IC_50_ value was estimated from the survival curve.

### Colony and sphere formation assays

For colony assay, one thousand miPSCs were cultured in 60 mm-dishes with miPS media with 1000U/ml LIF. After 24 h of seeding, media changed to miPS media without LIF supplemented with either 50% CM, 50% CM and 1 µM lapatinib, 50% CM and 5 µM lapatinib or with 50% CM and either 1,5 or 10 µM TX1-85-1. Cells were incubated at 37 °C with 5% CO_2_ for 7 days. After incubation, cells were fixed with 75% methanol and stained with Giemsa stain (Sigma-Aldrich, MO). The dishes were photographed and ImageJ software was used to count colonies with a diameter ≥ 50 µm. To assess cell’s ability to form spheroids, miPSCs cultured in miPS media supplemented with above conditions for one week were seeded at density of 1000 cells/well into 24-well ultra-low attachment plates (Prime Surface Dish, Sumitomo Bakelite, Japan) with serum-free media consisting of DMEM, 2 mM L-glutamine, 0.1 mM β-ME, 0.1 mM NEAA, and ITS-X (Wako, Japan) supplemented with penicillin/streptomycin with 0,1 or 5 µM lapatinib or 1, 5, 10 µM TX1-85-1. The cells were then incubated at 37 °C in 5% CO_2_ for 7 days and the formed spheres were photographed using the Olympus IX81 microscope equipped with a light fluorescence device (Olympus, Japan).

### Tube-formation assay

The miPSCs cultured in miPS media with either 50% CM, 50% CM and 1 µM lapatinib, or 50% CM and 5 µM lapatinib for one week were trypsinized and 7.5 × 10^4^ cells were resuspended in EBM2 media (Lonza, Switzerland). Cells were seeded in 24-well plates coated with Matrigel (Corning, NY) with EBM2 media supplemented with 1 µg/mL ascorbic acid, 22.5 µg/mL heparin, 0.2 µg/mL hydrocortisone, and 2% FBS and incubated at 37 °C in 5% CO_2_. After 24 h, images were taken with the Olympus IX81 microscope (Olympus, Japan) and analyzed with the angiogenesis analyzer tool in Image J (National Institutes of Health, MD).

### Small interfering RNA (siRNA)

For siRNA experiment, silencer predesigned siRNA targeting ErbB3 was purchased from (Thermo Fisher Scientific, USA). The oligonucleotide sequences of the siRNA were; sense 5′ CGAGAAUAUUCGCCCAACCtt 3′, antisense 5′ GGUUGGGCGAAUAUUCUCGtc 3′. The Negative control siRNA was from (Qiagen, USA). The miPSCs were transfected with Lipofectamine RNAiMAX (Thermo Fisher Scientific, USA) according to the manufacturer's instructions and cells were collected and subjected to western blotting.

### Western blotting

The miPSCs cultured in miPS media with either 50% CM, 50% CM and 1 µM lapatinib, or 50% CM with 5 µM lapatinib or with 50% CM and 10 µM TX1-85-1 for one week and cells transfected with either negative control siRNA or ErbB3’s siRNA were lysed with a lysis buffer consisting of 50 mM Tris pH 8.0 (Wako, Japan), 150 mM sodium chloride (Wako, Japan), 0.05% Triton X-100 (Sigma-Aldrich, MO), and 0.5 mM ethylenediaminetetraacetic acid (EDTA) (Wako, Japan). The protease and phosphatase inhibitors (Nacalai Tesque, Japan) were also added to the lysis buffer. The lysed cells were further sonicated, centrifuged at 15000xg for 30 min at 4 °C and supernatants were collected and kept at − 80 °C. The total protein concentration was assessed by Pierce™ BCA Protein Assay Kit (Thermo Fisher Scientific, MA) and 30 µg of total protein were loaded on SDS-PAGE, transferred to Immobilon-FL transfer membrane (PVDF, Merck Millipore, Germany). The membranes were blocked with 5% Skim Milk (Wako, Japan) and incubated at 4 °C overnight with primary antibodies against β-tubulin (Cell Signaling Technology, MA), β-actin (Wako, Japan), Erk1/2, phospho-Erk1/2 (Thr202/Tyr204), (Cell Signaling Technology, MA), Oct3/4 (Santa Cruz Biotechnology, TX), Nanog (GeneTex, CA) and CD24 (Thermo Fisher Scientific, MA). Then, membranes were washed with tris-buffered saline with 0.1% tween (Wako, Japan) and incubated with either anti-rabbit or anti-mouse HRP–conjugated secondary antibodies for one hour at room temperature. The blots were visualized with the Western Lightning Plus ECL Substrate (Perkin Elmer, MA) and imaged with WSE-6100 LuminoGraph I system (ATTO Corporation, Japan). The western blot band densitometry was calculated with ImageJ software.

## Supplementary Information

Supplementary Information 1.

Supplementary Information 2.

Supplementary Information 2.

Supplementary Information 4.

Supplementary Information 5.

Supplementary Information 6.

## Data Availability

All data generated or analyzed during this study are included in this article.
