# Supplementary figures and images for: The significance of ErbB2/3 in the conversion of induced pluripotent stem cells into cancer stem cells
Source: Sci Rep. 2022 Feb 17;12:2711. doi: 10.1038/s41598-022-04980-y (PMC8854581; doi:10.1038/s41598-022-04980-y)

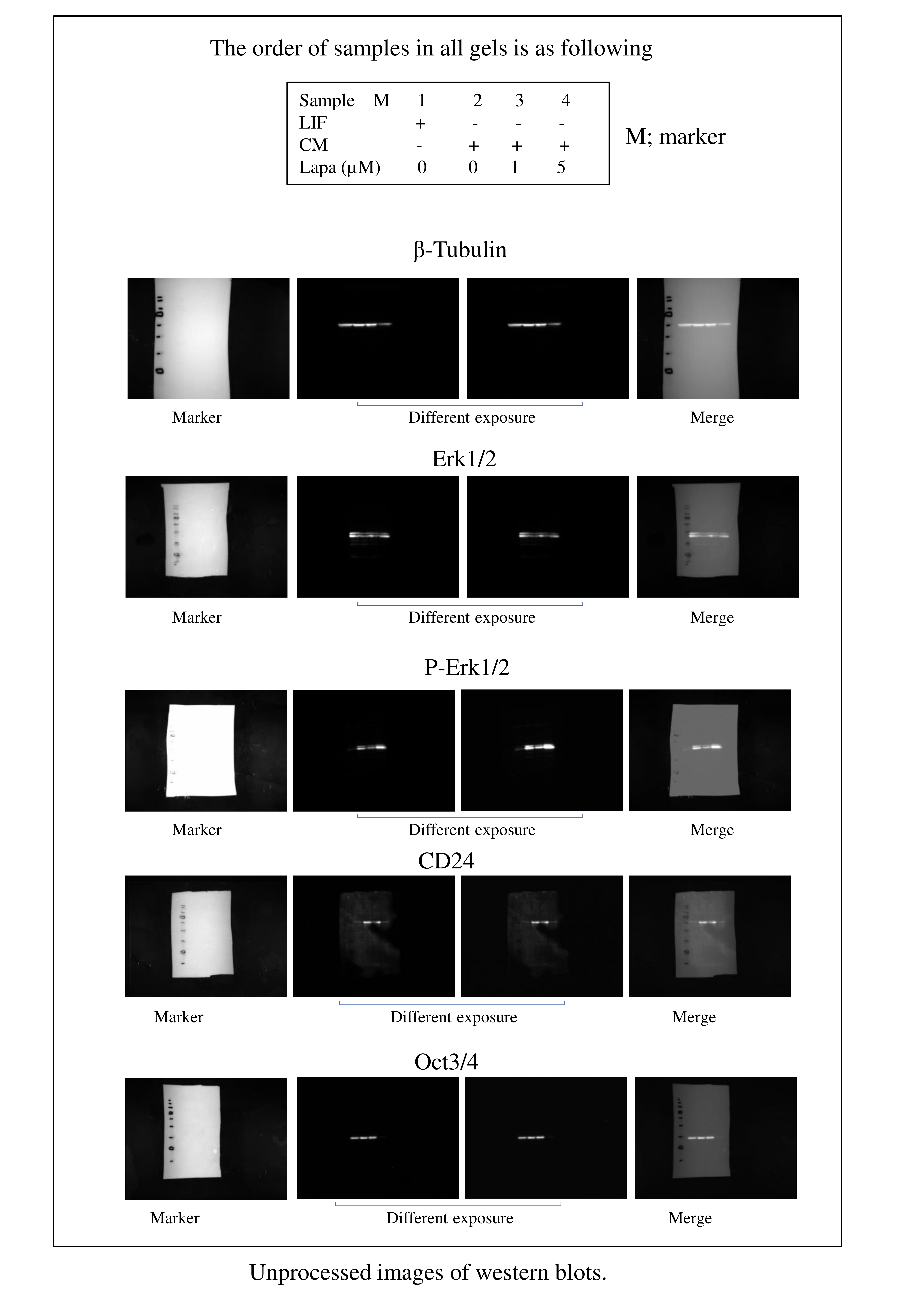

Supplement: Supplementary file 1 — Supplementary Information 1. [file 41598_2022_4980_MOESM1_ESM.jpg]

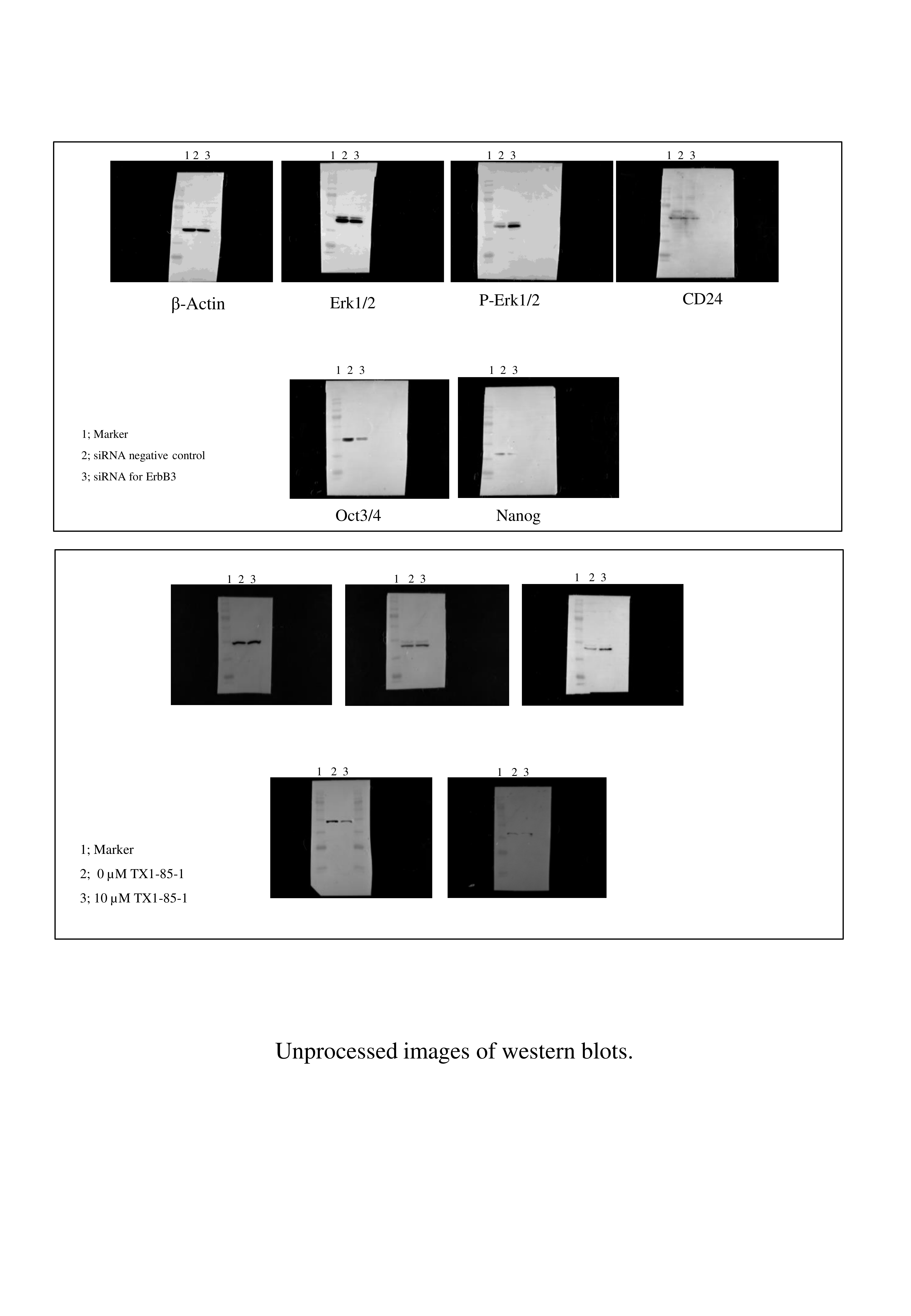

Supplement: Supplementary file 3 — Supplementary Information 2. [file 41598_2022_4980_MOESM3_ESM.jpg]
