# Supplementary material for: The significance of ErbB2/3 in the conversion of induced pluripotent stem cells into cancer stem cells
Source: Sci Rep. 2022 Feb 17;12:2711. doi: 10.1038/s41598-022-04980-y (PMC8854581; doi:10.1038/s41598-022-04980-y)
Supplement: Supplementary file 4 — Supplementary Information 4. [file 41598_2022_4980_MOESM4_ESM.docx]

**The significance of ErbB2/3 in the conversion of induced pluripotent stem cells into cancer stem cells**

Ghmkin Hassan^1,2^, Maram H. Zahra^1^, Akimasa Seno^1,3^, Masaharu Seno^1*^

1. Department of Biotechnology and Drug Discovery, Graduate School of Interdisciplinary Science and Engineering in Health Systems, Okayama University, Okayama 700-8530, Japan.
2. Department of Genomic Oncology and Oral Medicine, Graduate School of Biomedical and Health Science, Hiroshima University, Hiroshima 734-8553, Japan.
3. The Laboratory of Natural Food and Medicine, Co., Ltd., Okayama 700-8530, Japan

**Corresponding:**

Prof. Masaharu Seno,

Professor of Nano-Biotechnology, Department of Biotechnology and Drug Discovery

Graduate School of Interdisciplinary Science and Engineering in Health Systems, Okayama University

Office: 3.1.1 Tsushima-Naka, Kita, Okayama 700-8530, Japan.

Voice/Fax: +81-86-251-8216

E-mail: [mseno@okayama-u.ac.jp](mailto:mseno@okayama-u.ac.jp)

**Supplementary Table 1**. Transcript count number for genes of ErbB receptor family, their ligands and genes related to JAK-STAT, focal adhesion pathways.

| Transcript | Count number | | |
| --- | --- | --- | --- |
|  | miPSCs | miPS-PK8cm | miPS-PK8cmP |
| Erbb2 | 155 | 432 | 248 |
| Erbb3 | 78 | 318 | 355 |
| Erbb4 | 0 | 0 | 0 |
| Egfr | 12 | 59 | 77 |
| Nrg1 | 15 | 22 | 36 |
| Nrg2 | 14 | 228 | 115 |
| Nrg3 | 0 | 0 | 0 |
| Jak1 | 361 | 1029.762 | 839.12073 |
| Ccl2 | 0 | 112 | 1 |
| Il17d | 126 | 398.5962 | 197.35665 |
| Cxcl1 | 2 | 17.78859 | 19.788589 |
| Cxcl12 | 8 | 34.2595 | 2.3663867 |
| Ccl7 | 1 | 28.98881 | 0 |
| Itga3 | 847 | 1573.302 | 1851.461 |
| Itga5 | 767 | 1894.155 | 1344.1076 |
| Itga6 | 1106 | 3719.792 | 3346.5441 |
| Itgav | 150 | 405.8434 | 322.30187 |
| Itgb3 | 25 | 363.019 | 404.17885 |
| Itgb4 | 112 | 339.3009 | 367.26322 |
| Itgb5 | 473 | 1405.298 | 1050.2024 |
| Ptk2 | 447 | 666.7427 | 603.42861 |
| Src | 546 | 1712.316 | 1188.8727 |


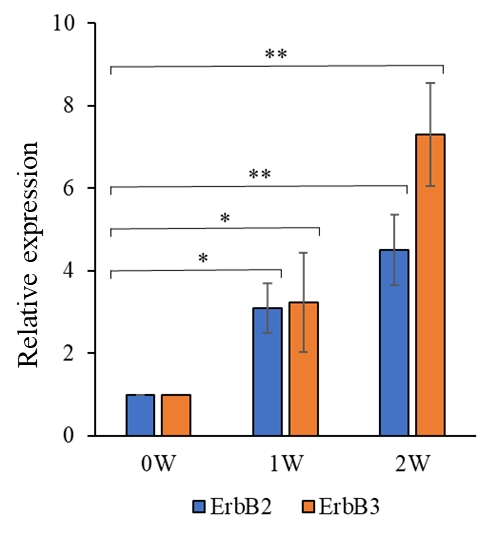


**Supplementary Figure 1**. Relative expression levels of ErbB2 and ErbB3 genes in week 0,1 and 2 of culturing miPSCs in CM assessed by RT-qPCR. Data were obtained from three different experiments. *, p < 0.05; **, p < 0.001. 0W, week 0; 1W, after one week of conversion; 2W, after two weeks of conversion.

**Supplementary Table 2**. Primer used in the study

| **Gene** | **Accession Number** | **Forward primer** | **Reverse primer** |
| --- | --- | --- | --- |
| ErbB2 | NM_001003817.1 | CGAGTGTCAGCCTCAAAACA | TCCGGGTACTTCCAGATAGG |
| ErbB3 | NM_010153.2 | ATGCGCCCATTTTCGTGATG | CTGGTCCCTTACACCCTTGG |
| Actb | NM_007393.5 | AAATCTGGCACCACACCTTC | GGGGTGTTGAAGGTCTCAAA |


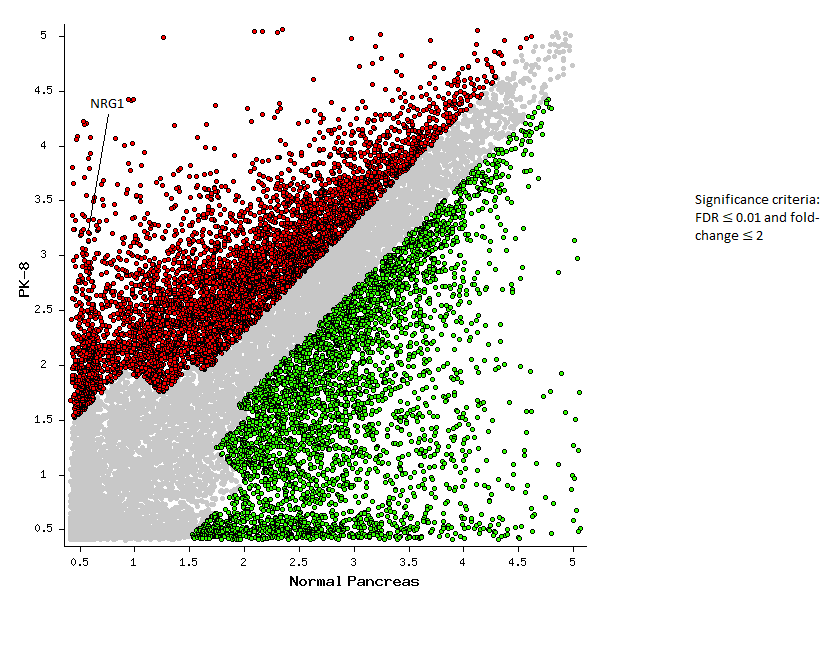


**Supplementary Figure 2**. Representative image for scatter plot of over- and down-expressed genes in PK8 cells compared with normal pancreas. Microarray data was obtained from GEO database. PK8 from GSE141247 dataset and normal pancreas from GSE71729 dataset. Red dots represent significant over-expressed genes in PK8 cells, and green dots are significant down-expressed genes. Significance criteria were false discovery rate (FDR) ≤ 0,0001 and fold change ≥ 2.
